# Health and Human Rights Concerns of Drug Users in Detention in Guangxi Province, China

**DOI:** 10.1371/journal.pmed.0050234

**Published:** 2008-12-09

**Authors:** J. Elizabeth Cohen, Joseph J Amon

**Affiliations:** 1 Independent Consultant, New York, New York, United States of America; 2 HIV/AIDS Program, Human Rights Watch, New York, New York, United States of America; University of Colorado, United States of America

## Abstract

**Background:**

Although confinement in drug detoxification (“detox”) and re-education through labor (RTL) centers is the most common form of treatment for drug dependence in China, little has been published about the experience of drug users in such settings. We conducted an assessment of the impact of detention on drug users' access to HIV prevention and treatment services and consequent threats to fundamental human rights protections.

**Methods and Findings:**

Chinese government HIV and anti-narcotics legislation and policy documents were reviewed, and in-depth and key informant interviews were conducted with 19 injection drug users (IDUs) and 20 government and nongovernmental organization officials in Nanning and Baise, Guangxi Province. Significant contradictions were found in HIV and antinarcotics policies, exemplified by the simultaneous expansion of community-based methadone maintenance therapy and the increasing number of drug users detained in detox and RTL center facilities. IDU study participants reported, on average, having used drugs for 14 y (range 8–23 y) and had been confined to detox four times (range one to eight times) and to RTL centers once (range zero to three times). IDUs expressed an intense fear of being recognized by the police and being detained, regardless of current drug use. Key informants and IDUs reported that routine HIV testing, without consent and without disclosure of the result, was the standard policy of detox and RTL center facilities, and that HIV-infected detainees were not routinely provided medical or drug dependency treatment, including antiretroviral therapy. IDUs received little or no information or means of HIV prevention, but reported numerous risk behaviors for HIV transmission while detained.

**Conclusions:**

Legal and policy review, and interviews with recently detained IDUs and key informants in Guangxi Province, China, found evidence of anti-narcotics policies and practices that appear to violate human rights and imperil drug users' health.

## Abstract 2

Based on their review of Chinese government legislation and policy documents, and using interviews with recently detained injection drug users and officials in Guangxi Province, Elizabeth Cohen and Joseph Amon find evidence of antinarcotics policies and practices that may compromise the health and human rights of drug users.

## Editors' Summary

**Background.:**

Ever since the AIDS (acquired immunodeficiency syndrome) epidemic began, needle sharing by injection drug users (IDUs) has been a major transmission route for the human immunodeficiency virus (HIV), the blood-borne virus that causes AIDS. In China, for example, the AIDS epidemic began in earnest in 1989 when 146 HIV-positive IDUs were identified in Southwest Yunnan. By 1998, HIV infections had been reported throughout China, 60%–70% of which were in IDUs. These days, nearly half of new HIV infections in China are associated with injection drug use and 266,000 of the 700,000 HIV-positive people in China are drug users. Faced with these figures, the Chinese government has recently introduced measures to reduce HIV transmission among the estimated 3–4 million IDUs in China. These measures include increased provision of methadone maintenance treatment clinics and needle exchanges and the establishment of HIV prevention programs that target IDUs.

**Why Was This Study Done?:**

Alongside these progressive public-health practices, China has extremely punitive anti-narcotics policies. IDUs are routinely confined without legal review in drug detoxification centers or sent to re-education through labor (RTL) centers, sometimes for many years. In 2005, these centers housed more than 350,000 drug users yet little is known about the conditions in these centers or how the Chinese anti-narcotic policy affects human rights or access to HIV prevention and treatment services. In this study, the researchers investigated these issues by interviewing IDUs and “key informants” (government officials and members of nongovernmental organizations [NGOs] who provide services to IDUs) about their experiences of detoxification centers and RTL centers in two cities in Guangxi Province, China.

**What Did the Researchers Do and Find?:**

The researchers recruited 19 IDUs who had been recently confined in a detoxification center or RTL center and 20 key informants (including a doctor at a detoxification center and a former RTL center guard). In the interviews the researchers used a semi-structured questionnaire to ask the participants about their experiences of detoxification centers and RTL centers. All the IDUs reported that they were repeatedly tested for HIV while in detention but never given their test results even when they asked for them. Key informants confirmed that repeated HIV testing without result disclosure is the current policy in detoxification centers and RTL centers. All the IDUs expressed concerns about inadequate access to health care in detention. In particular, most of the IDUs who were taking antiretroviral drugs before detention were unable to continue their treatment during detention, although two received antiretroviral drugs by negotiating with their guards. The IDUs and key informants also both noted that very little information or means of HIV prevention was provided in the detoxification centers and RTL centers and that HIV-related risk behaviors, including injection drug use and unsafe sex, occurred in both types of center. Finally, the IDUs reported that their fear of being recognized by the police and detained even if not taking drugs prevented them from seeking HIV tests, HIV treatment, and help for their drug addiction.

**What Do These Findings Mean?:**

This study has several limitations in addition to its small size. For example, because the IDUs were self selected—they responded to posters asking if they would be interviewed—their views may not be representative of all IDUs. Similarly, the key informants who were interviewed might have had different opinions from those who chose not to participate. Furthermore, the results reported here cannot be generalized to other areas of China. Nevertheless, the consistent experiences reported by the IDUs and confirmed by the key informants suggest that China's anti-narcotic policies and practices violate the human rights of IDUs and put their health in danger by making it hard for them to access HIV prevention and treatment or adequate treatment for their drug addiction. This situation, if not remedied, is likely to jeopardize China's attempts to control its HIV epidemic.

**Additional Information.:**

Please access these Web sites via the online version of this summary at http://dx.doi.org/10.1371/journal.pmed.0050234.

- This study is further discussed in a *PLoS Medicine* Perspective by Steve Koester
- Avert, an international AIDS charity, provides information on all aspects of HIV/AIDS, including HIV and AIDS in China and HIV prevention, harm reduction, and injecting drug use
- The UNAIDS 2008 Country Progress Report provides up-to-date details about the AIDS situation in China
- HIVInSite provides links to more information about HIV/AIDS in China.
- Human Rights Watch works on health and human rights and human rights developments in China
- The US National Institute on Drug Abuse provides a booklet entitled Principles of Drug Addiction Treatment: A Research Based Guide (in English and Spanish)
- UN Office on Drugs and Crime has information on HIV/AIDS in prisons

## Introduction

Among an estimated five to six million illegal drug users, China is believed to have between three and four million individuals who inject drugs, primarily heroin [1]. Injection drug use is a major route of transmission for new HIV infections in China, and at the end of 2007 an estimated 266,000 drug users were living with HIV/AIDS and nearly half of all new HIV infections were believed to be associated with injection drug use [2].

The Chinese government's response to the HIV/AIDS and injection drug use epidemics has been characterized as belated but “bold” [3]. In the past few years, the government has announced dramatic programs to address drug dependency and HIV, greatly expanding methadone maintenance therapy and HIV prevention programs targeting injection drug users (IDUs), including IDUs confined to detoxification (“detox”) and re-education through labor (RTL) centers [4–7]. China's 1998–2010 Strategic Plan for HIV/AIDS included a specific goal to provide, by 2002, “health education on preventing HIV/AIDS and STDs at all detox centers and re-education centers as well as in 80% of jails” [5]. The government has dedicated increasing resources, supplemented by international funding, towards these goals [1,8].

At the same time, the government has adopted increasingly punitive ant-narcotic policies. Between 1995 and 2000, China quadrupled its capacity to provide compulsory detoxification, [9] and in 2005 the government launched a “National People's War on Illicit Drugs” with the goal of further increasing the number of people detained [10,11]. The most recently available data from 2005 indicate that there were approximately 700 mandatory drug detox centers in China and 165 RTL centers housing a total of more than 350,000 drug users [12,13]. According to government policy, first-time offenders are sent to a drug detox center for 3–6 mo, and repeat offenders are sentenced to a RTL center for 1–3 y [14]. In practice, multiple sentences to detox centers are common. While guidelines specify that drug detoxification sentences may not exceed 1 y, local authorities may hold an inmate indefinitely without official review because detainees are not processed through the legal system and have severely limited due process rights [14].

The paradox of progressive public health practices and punitive antinarcotics policies, and ongoing tensions between the two, was highlighted in the government's most recent regulations on drug policy. In 2007 the Standing Committee of the National People's Congress passed a new antidrug law, intended to go into effect in June 2008 [15]. The law gives police broader power to conduct drug searches, but also supports community-based treatment. While eliminating the use of RTL centers for the detention of drug users, the law increases the period of detention to up to 6 years, with a new category of detention, “compulsory isolation detoxification” (qiangzhi geli jiedusuo), with 1- to 3-y sentences followed by up to 3 y of community rehabilitation [16]. Authority over the new detention centers, and the definition of community rehabilitation, have not yet been clearly described.

To investigate the experience of IDUs in detention in relation to HIV prevention and treatment services, we reviewed HIV and antinarcotics legislation and policy and conducted interviews with IDUs and government and nongovernmental organization (NGO) key informants in two cities in southern China.

## Methods

The research was conducted in the cities of Baise and Nanning in Guangxi Province in southern China and was composed of three distinct approaches: (1) review of Chinese government HIV and antinarcotics legislation and policies; (2) key informant interviews with government and NGO officials working in HIV and antinarcotics programs; and (3) in-depth interviews with IDUs who had recently been detained in detox or RTL centers.

### Study Site

Baise is a city of 325,000 people that in 2004 had 210 people registered as drug users and 84 new HIV infections, 74% of which were believed to be related to injection drug use [17]. Local government officials estimated that there were currently 466 registered drug users, and that 67% of drug users in Baise are HIV infected. A study conducted in 1997 in Baise found 77% of drug users infected [18].

Nanning, the provincial capital, has approximately one million urban residents, and an estimated 5,000 registered drug users [17]. According to key informants, two detox centers house an estimated 1,000 drug users annually, and three RTL centers—one for women, one exclusively for drug users, and one with a mix of drug and nondrug-related residents—house an estimated 3,000 drug users.

### Legal and Policy Review

Past and current HIV and antinarcotics legislation, regulation, and policy documents in English and Chinese were reviewed. The documents included national, regional, and local policy documents from government and nongovernmental organizations, and reports and proposals to UN and multilateral donors by the Chinese government. In addition, local news reports of HIV and antinarcotics campaigns were reviewed.

### Key Informant Interviews

Interviews with key informants from NGOs and the Chinese government were conducted both prior to and following interviews with IDUs to identify salient issues and probe specific findings related to HIV and antinarcotics policy and practice. Interviews with health care providers and NGO key informants emphasized access and barriers to the provision of HIV prevention and treatment services to IDUs in community and detention settings, while interviews with government key informants emphasized official policies and practices.

### IDU Interviews

Organizations providing services to IDUs in both cities permitted the posting of a notice seeking IDUs interested in being interviewed. IDUs responding to the notice were screened for eligibility—a history of current or past injection drug use and recent (<5 y) confinement to mandatory detoxification or RTL center—and asked to consent to open-ended interviews. Respondents were asked to refer other IDUs. Recruitment ended when responses to our notices and referrals diminished and consistency of reporting across key domains was achieved [19,20]. All interviews took place in July 2007.

Interviews were conducted in Mandarin Chinese using a semistructured survey instrument by one author (JEC). Interviews covered a range of domains and emphasized IDU experiences in detox and RTL centers, including general living conditions, health status, drug use while detained, exposure to HIV prevention information and services, and access to health care. IDUs were also asked about their experiences prior to and following confinement in detox and RTL centers, including knowledge of HIV prevention and exposure to HIV prevention services, access to health care, and contact with police. Reimbursements were provided to IDUs for transportation to the interview location. Additional and extended testimony from IDUs are presented elsewhere [21].

Each interview lasted approximately 3 h and was tape recorded, translated into English, and transcribed. Interview data were hand-coded and the authors conducted a content analysis to identify key themes corresponding to the interview guide, as well as emergent topics. In the first analysis of the data an initial set of codes was generated to capture key constructs. Subsequent analyses were undertaken to examine the consistency of reports across themes and examine negative evidence [19].

### Ethical Review

All participants provided oral informed consent to participate and were assured anonymity, including key informants who uniformly requested it as a precondition for providing information. Interviews were conducted in private and study participants were assured that they could end the interview at any time or decline to answer any question(s) without consequence. The research approach, and ethical and human participant protections associated with the research, were reviewed and approved prior to conducting the research by legal, programmatic, and policy staff at Human Rights Watch. We did not seek ethical approval from Chinese authorities as we felt that this would have endangered, not protected, the safety of the participants.

Because the ethics review for this study was conducted by an internal committee, and to assess the adequacy of measures taken to assure the protection of study participants, the *PLoS Medicine* editors asked the journal's Advisory Committee on Competing Interests and Publication Ethics to review these aspects of the study. The *PLoS Medicine* editors and a majority of the committee recommended publication, and found that the protection of the confidentiality and safety of the participants, as described, was sufficient. However, the lack of independent, external review of the protocol was found to be less than ideal. Text S1 provides additional information about the review process undertaken by the authors.

## Results

A total of 20 key informant interviews were conducted with individuals from the Chinese Center for Disease Control (CDC), provincial and city health departments, domestic and international NGOs, including medical staff at methadone and AIDS clinics, a detoxification center, and a former RTL center guard. Key informants provided access to legal and policy documents regarding Chinese government HIV and antinarcotics efforts which were complemented by public records searches and news reports.

Twenty injection drug users directly contacted, or were referred to, interviewers. Nineteen (15 male and four female) IDUs had been recently released from mandatory detoxification or RTL centers and were determined to be eligible for interview. Eight were residents of Baise and 11 were from Nanning. Drug users ranged in age from 28 to 45 y and reported, on average, having used drugs for 14 y (range 8–23 y) and having been confined to mandatory detox centers four times (range one to eight) and to RTL centers once (range zero to three). Six of the 19 IDU had been last released from detox or RTL centers in 2007, seven in 2006, and six in 2005 or earlier. Twelve IDUs reported their serostatus as HIV-infected, seven were unaware of their serostatus, and none reported their status as HIV-negative.

### Fear of Arrest and Access to Services

In 2007 the Chinese government launched an antinarcotics campaign entitled “Wind and Thunder Sweeping Narcotics” that strengthened regulations permitting random urine tests for registered drug users [11]. Similar to the neighborhood watch initiatives that have been a staple of Chinese law enforcement for the last century, the campaign provided monetary incentives to citizens for reporting neighbors, relatives, and community members of using drugs. All IDUs in our study expressed an intense fear of being recognized by the police (from past contact, or from resembling a “drug user”), or reported by neighbors and relatives, and detained.

In both Baise and Nanning, all IDUs interviewed believed that whether IDUs were fined, sent to a detox center or a RTL center depended upon arrest quotas and occupancy rates in different facilities. One respondent stated: “If you get arrested when they have enough people in RTL center then you are safe, but if you get arrested at another time, then you can be put into RTL center.”

IDUs and key informants explained that during “high profile” periods, such as the period preceding the June 26 International Day Against Drug Abuse and Illicit Trafficking, IDUs were frequently picked up by police based upon their past record and sent to detoxification, even if they were not currently using drugs. During these periods, most IDUs reported rarely leaving home.

IDUs interviewed described in various ways how the threat of arrest and/or detention impede IDUs from accessing HIV testing, clean needles, and methadone therapy. One IDU who did not know his serostatus said, “Sometimes I'm afraid I might be sick with AIDS but I'd rather be sick and free than go to get tested, get arrested and be sick in detox or RTL center.”

Almost all IDUs reported that police routinely conduct surveillance of pharmacies and methadone clinics and some IDUs reported having been arrested when seeking to buy clean needles or access methadone. A provincial government worker explained: “Part of the point of methadone centers is that it provides a way to keep control of drug users.” A former IDU reported that as he was leaving a government-run HIV testing site in Nanning, he was spotted by the police. He explained, “I had just come from having my blood drawn and police saw that my arm had an open mark and some blood. They stopped me and put me in detox.”

Despite these challenges some IDUs reported using methadone and all IDUs interviewed were aware of its availability. A government health official stated that training programs with police had been initiated to facilitate the coexistence of law enforcement objectives with harm reduction programs, and that police, in general, had become more tolerant of methadone programs.

### Judicial Oversight

Chinese law specifies that all aspects of detention are left to the discretion of local officials and prescribes severely limited due process protections for people who are charged as drug users [14]. Some IDUs reported frustration that they were unable to challenge their detentions or appeal for review of the sentence or conditions where they were held. One IDU interviewed said, “We are taken off the street, brought to the police station and put into detox or RTL center, for as long as three years. The police can do whatever they want with us. There are no laws protecting our rights.”

### Conditions in Detention

IDUs who were interviewed described the conditions at RTL and detox centers in relation to HIV testing, access to health care, and HIV infection risk in almost identical terms. Some aspects of living conditions in different facilities, such as forced labor, and treatment by individual guards, varied.

#### Mandatory HIV testing.

Once detained in either detox or RTL centers, IDUs uniformly reported that they were repeatedly tested for HIV but never provided the result. IDUs were instructed to follow up with the local CDC upon release. Some IDUs reported trying to obtain HIV test results or serostatus information from facility guards and other personnel. One IDU said, “When we were in detox we asked the guards if we had AIDS and they said, ‘oh it doesn't matter, you won't die that fast from AIDS'.”

An HIV-infected former detainee said, “I was tested in detox twice for HIV, most recently in 2006, but was never told the result. Then when I got out I was so sick that I went to the clinic. They tested me and told me I have AIDS.”

A key informant who had urged China CDC officials to disclose the results of HIV tests to IDUs in detention said that government officials were afraid that “informing people will cause chaos in detox and too high a demand on services.” Key informants from the government, as well as sources from NGOs and among health care providers confirmed that repeated testing without disclosing the results is the current policy for detox, RTL centers, and prisons throughout the country. A former RTL center guard said that the guards used the HIV testing data “to know which female inmates they could sleep with without using a condom.”

#### Access to health care.

Although regular HIV testing was reported, all 19 drug users expressed concern about inadequate access to medical monitoring or health care. One HIV-infected IDU reported “I was very worried I was going to die the last time I was in detox. There was not enough food to eat and no one was checking my CD4 count.” A similar situation was reported by IDU study participants regarding RTL centers: “There is no CD4 count at RTL center. I knew I was getting sicker but I couldn't leave because I could still work.”

All of the IDUs interviewed reported that on-site medical personnel rarely treat anything beyond very basic ailments, and that any medications prescribed must be paid for by the detainee. A detox doctor interviewed corroborated this, saying, “If an inmate is very sick we take him to the hospital. If he doesn't have enough money the hospital won't accept him. What happens to the inmate at that point is not our responsibility.” None of the IDUs reported access to counseling or opiate substitution therapy to address their drug dependency.

#### Disruption of antiretroviral therapy.

Most IDU study participants who were on antiretroviral therapy prior to being sent to detox or RTL centers reported that they were unable to continue therapy while institutionalized. One IDU, who was released from detox in June 2007 said, “I started taking antiretroviral drugs (ARVs) before I was put into detox. Then when I was in [detox] I had to stop. I was really worried about my health but there was nothing I could do.”

Two IDUs reported receiving ARVs in detox, despite the lack of medical supervision, because a local NGO provided them with a monthly supply of ARVs, which they stockpiled in their cells. Both reported that their ability to access these drugs was dependent on their relationship with guards. One commented, “If you have a good relationship with a guard and they know you have AIDS and are taking ARVs they may be willing to let the medicine in.” An NGO official stated: “people are not treated for AIDS when they are in detox. Twenty people in a cell is not a good environment for supervising treatment.”

Government officials and key informants in local and international NGOs providing services to drug users confirmed that the continuity of access to ARVs was made on a case-by-case basis and largely dependent on negotiations with individual guards.

#### HIV infection risk.

IDUs uniformly reported that they received little or no information on HIV prevention while in detox or RTL centers. A former detainee from a detox center in Nanning said, “People come to talk about AIDS prevention one day a year. It's the same day they bring in the TV cameras and the nice food.”

NGO representatives agreed that beyond such high-profile days there were significant barriers to getting access to detainees in order to conduct HIV prevention programs. Like the provision of ARVs, their access depended solely on the cooperativeness of individual staff members at the centers.

All IDUs interviewed reported that HIV-related risk behaviors including injection drug use and unsafe sex existed in both detox and RTL centers. However, IDU participants reported differing degrees of availability of illegal drugs and some said that accessing drugs had become more difficult in recent years. Almost all IDUs said that when drugs were available, inmates shared needles, makeshift or otherwise, due to the lack of access to clean injection equipment.

The former RTL center guard interviewed said that sexual relations between guards and female detainees were common, and that guards supplied drugs to detainees in exchange for sex. He stated: “Women in RTL center need comforting, especially the younger ones. I would sleep with them to comfort them and then give them some heroin to make them feel better.”

#### General living conditions.

IDUs interviewed reported that the cells in detox centers in Baise and Nanning were approximately 15 by 15 feet (4.6 by 4.6 meters), and shared by as many as 30 people. In Baise, IDUs who had been in detox centers reported that they had been kept locked in their cells for most of the day, while IDUs in detox in Nanning or in RTL centers in either city reported being obligated to perform unpaid factory work, which they characterized as “slave labor.” These IDUs consistently reported that they were required to work long hours, from 7 a.m. until as late as 2 a.m., 7 d per week, and said that if they did not finish their work they were punished. Punishments could include having food withheld, not being allowed to sleep, or being beaten.

##  Discussion

Despite substantial progress by the Chinese government in adopting evidence-based HIV policies, and scaling up the overall response to HIV prevention by targeting IDUs, our research suggests that government antinarcotics campaigns and the confinement of drug users in detox and RTL centers in Guangxi Province are in opposition to the intent of expanded programs of information and treatment for drug users, and appear to violate their human rights and imperil their health and lives.

Illegal drug use in China is considered a violation of administrative law, which dictates that “drug takers must be rehabilitated” [22]. Chinese law further mandates that all patients in compulsory rehabilitation centers be provided with “medical and psychological treatment, legal education and moral education” [15]. Yet, neither detox nor RTL centers—the most common forms of “treatment” for drug use in China [23]—appear to provide effective rehabilitation from drug dependence. The government of China has established no standard for the provision of drug dependency treatment, and behavioral intervention or sustained substitution therapy provided in either detox or RTL centers are rare [24]. While some facilities may use methadone, buprenorphine, clonidine, lofixidine, and/or traditional Chinese medicine to assist with detoxification in institutional settings [25], it is commonly reported that drug users receive either no medicine or only herbal remedies [26,27], and access to medicine is dependent on the views of local authorities and the budget of the facility and the drug user.

Liu et al., in their study of behavioral change of Chinese IDUs who have spent time in detox centers reported that 95% of IDUs relapsed within 1 y of leaving detox [28]. They found no correlation between confinement at a drug detoxification center and drug use, and concluded that detox and RTL centers offer, at best, only a period of abstinence from drug use. Other studies have found similarly high relapse rates among those who are subjected to detox and RTL centers [29–32], and it is widely recognized that detoxification treatment alone is unlikely to be effective [33–36]. Furthermore, research in southern Guangxi Province found that increased police targeting of drug users and fear of confinement in detox and RTL centers had led to decreased use of peer education and needle and syringe exchange programs [24].

The assignment to, and confinement in, detox and RTL centers represent severe violations of international human rights laws, including: the detention of drug users without judicial oversight, the failure to provide adequate medical care and HIV prevention services, forced labor, and sexual abuse of female detainees. The UN Special Rapporteur on Torture has stated that the RTL center can “be considered a form of inhuman or degrading treatment or punishment, if not mental torture” and recommended that China abolish the centers [37]. IDUs participating in our study reported strikingly similar experiences in detention and in the community, where antinarcotics policies significantly increased their vulnerability to HIV infection, prevented their access to care, and failed to provide them with effective drug dependence treatment.

China has signed, but not yet ratified, the International Covenant on Civil and Political Rights (ICCPR), which means that China can not take retrogressive steps in relation to the treaty, nor violate it in spirit [38]. Article 9 of the ICCPR provides that any person “deprived of his liberty by arrest or detention shall be entitled to take proceedings before a court, in order that that court may decide without delay on the lawfulness of his detention and order his release if the detention is not lawful” [39]. The UN Human Rights Committee has interpreted this provision to apply to all deprivations of liberty, including noncriminal detention for drug dependency [40]. The UN Body of Principles for the Protection of All Persons Under Any Form of Detention, passed by the UN General Assembly in December 1988, similarly requires that persons “not be kept in detention without being given effective opportunity to be heard promptly by a judicial or other authority” [41].

International human rights law clearly affirms that, with the exception of the right to liberty, prisoners retain fundamental rights and freedoms subject to the restrictions that are unavoidable in a closed environment [42]. Prisoners, like all other persons, enjoy the right to the highest attainable standard of health, and the right to absolute protection against torture and cruel, inhuman, or degrading treatment or punishment [39,43–46]. While international law permits convicted criminals to be required to work as part of their punishment, drug users in mandatory detoxification centers have not been convicted of a crime in a court of law, and are therefore not covered by this provision [39].

The Chinese government's response to HIV/AIDS has evolved from denial to disregard to its current, increasingly pragmatic and evidence-based approach to policy-making. That evolution has been stimulated by quiet diplomacy, harsh public criticism, and recognition by Chinese officials of global standards and norms of public health practice. The government's response has reflected sensitivity to public health arguments and international pressure on human rights [47]. Nonetheless, the adoption and implementation of effective HIV and antinarcotics policies that are consistent with international human rights standards remains a struggle, often reflecting tension between national policies and local authorities. China's rapid expansion of HIV prevention and treatment for IDUs and increasingly repressive antinarcotics policy exemplifies this conflict.

There are several limitations to this research. The recruitment of drug users required nonrepresentative sampling, and their reports of their experiences in detox and RTL centers may not be generalizable to all drug users. For example, drug users participating in the study had relatively long histories of drug use and contact with police, and their experiences may be different from those of younger drug users. Many government and NGO officials were unwilling to comment without higher level authorization. Those who did agree to be interviewed agreed to speak only without attribution, and may have presented a different perspective than those who chose not to participate. Generalizing our results beyond Nanning and Baise is difficult because drug users and key informants reported that conditions at different detox and RTL center facilities vary, and even within one facility individual guards adopt different policies with different detainees. While the 19 IDUs we spoke with constitutes a small sample, they reported consistently similar experiences prior to detention and while in detox and RTL centers. Key informants from NGOs, Chinese health care workers, and a RTL center guard confirmed the accuracy of the reports provided by IDUs.

### Conclusion

To our knowledge, this study presents the first account in the English-language peer-reviewed literature of the experience of drug users in detoxification centers and RTL centers in China. The Chinese government has received international praise for adopting a serious approach to the HIV epidemic. Our research in Guangxi Province, however, has raised concerns that increasingly repressive antinarcotics policies and confinement in detoxification and RTL centers not only violate human rights but deny users access to basic health services and could imperil drug users' health. The failure of the Chinese government to ensure that drug users in detention receive effective treatment for drug dependency and have access to HIV prevention and treatment services while in detox or RTL centers constitutes a serious risk to the right to life, and jeopardizes the success of China's HIV goals.

## Supporting Information

Text S1 Additional Information on the Ethical and Human Subjects Review of the Study Protocol (58 KB PDF)

## Abbreviations

IDU: injection drug user
NGO: nongovernmental organization
RTL: re-education through labor

## References

[pmed-0050234-b001] Country Coordinating Mechanism of China (2004). Reducing HIV transmission among and from vulnerable groups and alleviating its impact in seven provinces in China. Round four proposal to The Global Fund against HIV, TB and malaria.

[pmed-0050234-b002] People's Republic of China (2008). UNGASS Country Progress Report, January 2006–December 2007.

[pmed-0050234-b003] Wu Z, Sullivan SG, Wang Y, Rotheram-Borus MJ, Detels R (2007). Evolution of China's response to HIV/AIDS. Lancet.

[pmed-0050234-b004] Sullivan SG, Wu Z (2007). Rapid scale up of harm reduction in China. Int J Drug Policy.

[pmed-0050234-b005] State Council of the People's Republic of China (1998). Chinese National medium-and long-term strategic plan for HIV/AIDS prevention and control (1998–2010). State Council Document GF.

[pmed-0050234-b006] State Council of the People's Republic of China (2001). China's action plan for reducing and preventing the spread of HIV/AIDS (2001–2005). State Council Document.

[pmed-0050234-b007] State Council of the People's Republic of China (2004). State council notice on strengthening HIV/AIDS prevention and control. State Council Document 2004(7).

[pmed-0050234-b008] The Bill & Melinda Gates Foundation (2007 November 14). Major commitment to expand HIV prevention in China.

[pmed-0050234-b009] Reid G, Costigan G (2002). Revisiting ‘the hidden epidemic': a situation assessment of drug use in Asia in the context of HIV/AIDS.

[pmed-0050234-b010] National Narcotics Control Commission (2006). Annual Report on Drug Control in China [in Chinese].

[pmed-0050234-b011] Xinhua News Agency (2007 September 18). Beijing introduces compulsory urine tests to keep drug addicts clean.

[pmed-0050234-b012] He Y, Swanstrom N (2006). China's war on narcotics: Two perspectives.

[pmed-0050234-b013] Xinhua News Agency (2004 June 21) China registers 740,000 drug addicts.

[pmed-0050234-b014] State Council of the People's Republic of China (1995). Methods for forced detoxification [in Chinese].

[pmed-0050234-b015] Standing Committee of the 10th National People's Congress (2007). Prohibited drugs law of the People's Republic of China [in Chinese].

[pmed-0050234-b016] Yu W (2007). New anti-drug law uses quarantine to replace controversial re-education through labor..

[pmed-0050234-b017] China National Knowledge Infrastructure (2008). Database [in Chinese]..

[pmed-0050234-b018] Yu XF, Chen J, Shao Y, Beyrer C, Lai K (1998). Two subtypes of HIV-1 among injection-drug users in southern China. Lancet.

[pmed-0050234-b019] Auerbach CF, Silverstein LB (2003). Qualitative data: An introduction to coding and analysis.

[pmed-0050234-b020] Bailey K (1994). Methods of social research.

[pmed-0050234-b021] Human Rights Watch (2008). An unbreakable cycle: Drug dependency, mandatory confinement and HIV/AIDS in Guangxi Province, China. New York: Human Rights Watch.

[pmed-0050234-b022] State Council of the People's Republic of China (2000). White paper on narcotics control.

[pmed-0050234-b023] Tang Y, Zhao D, Zhao C, Cubells J (2006). Opiate addiction in China: Current situation and treatments. Addiction.

[pmed-0050234-b024] Hammett T, Bartlett N, Chen Y, Ngu D, Cuong N (2005). Law enforcement influences on HIV prevention for injection drug users: Observations from a cross-border project in China and Vietnam. Int J Drug Policy.

[pmed-0050234-b025] Zhao C, Liu Z (2004). Drug abuse in China. Ann N Y Acad Sci.

[pmed-0050234-b026] McDonald H (2005 June 25). Chinese addicts get cold turkey and herbs. The Age..

[pmed-0050234-b027] Human Rights Watch (2003). Locked doors: The human rights of people living with HIV/AIDS in China.

[pmed-0050234-b028] Liu H, Grusky O, Zhu Y, Li X (2006). Do drug users who frequently receive detoxification treatment change their risky drug use practices and sexual behavior. Drug Alcohol Depend.

[pmed-0050234-b029] Zhou Y, Li X (1999). Demographic characteristics and illegal drug use patterns among attendees of drug cessation programs in China. Subst Use Misuse.

[pmed-0050234-b030] Wu Z (1999). Preventing HIV infection among injection drug users in China. Global Research Network, HIV Prevention in Drug-Using Populations, Second Annual Meeting Report.

[pmed-0050234-b031] United Nations Drug Control Program (2000). China country profile.

[pmed-0050234-b032] The Joint United Nations Programme on HIV/AIDS, United Nations International Drug Control Program (2000). Drug use and HIV vulnerability: Policy research study in Asia.

[pmed-0050234-b033] United Nations Office of Drugs and Crime (2002). Contemporary drug abuse treatment: A review of the evidence base.

[pmed-0050234-b034] National Institute of Drug Abuse (1999). Principles of effective drug addiction treatment: A research-based guide..

[pmed-0050234-b035] Center for Substance Abuse Treatment (2002). Tip 45: Detoxification and substance abuse treatment.

[pmed-0050234-b036] Schottenfeld RS, Chawarski MC, Mazlan M (2008). Maintenance treatment with buprenorphine and naltrexone for heroin dependence in Malaysia: A randomised, double-blind, placebo-controlled trial. Lancet.

[pmed-0050234-b037] United Nations Commission on Human Rights (2006). Report of the Special Rapporteur on torture and other cruel, inhuman or degrading treatment and punishment: Mission to China. E/CN.4/2006/6/Add.6, paras 64, 82..

[pmed-0050234-b038] United Nations (1969). Vienna Convention on the Law of Treaties. United Nations Treaty Series, vol. 1155.

[pmed-0050234-b039] United Nations (1966). International covenant on civil and political rights. UN Doc. A/6316 (1966).

[pmed-0050234-b040] United Nations (1994). General Comment 8, Article 9, Sixteenth session, 1982. United Nations, Compilation of General Comments and General Recommendations Adopted by Human Rights Treaty Bodies, UN Doc. HRI/GEN/1/Rev.1(8).

[pmed-0050234-b041] United Nations (1988). Body of principles for the protection of all persons under any form of detention or imprisonment.

[pmed-0050234-b042] United Nations (1992). Humane treatment of persons deprived of liberty. Human Rights Committee General Comment 21. Compilation of General Comments and General Recommendations Adopted by Human Rights Treaty Bodies, UN Doc. HRI/GEN/1/Rev.

[pmed-0050234-b043] United Nations (1984). Convention against torture and other cruel, inhuman or degrading treatment or punishment. UN Doc. A/39/51.

[pmed-0050234-b044] United Nations (1990). Basic principles for the treatment of prisoners. UN General Assembly Resolution 45/111, Article 9.

[pmed-0050234-b045] World Health Organization, The Joint United Nations Programme on HIV/AIDS (1999). Guidelines on HIV infection and AIDS in prisons. UNAIDS Doc 99.47/E.

[pmed-0050234-b046] Committee on Economic, Social, and Cultural Rights (2000). General comment No. 14: The right to the highest attainable standard of health. UN Doc E/C.12/2000/4.

[pmed-0050234-b047] Amon J, Worden M (2008). High hurdles for health. China's Great Leap.

