# Supplementary material for: Health and Human Rights Concerns of Drug Users in Detention in Guangxi Province, China
Source: PLoS Med. 2008 Dec 9;5(12):e234. doi: 10.1371/journal.pmed.0050234 (PMC2596857; doi:10.1371/journal.pmed.0050234)
Supplement: Supplementary file 1 [file pmed.0050234.sd001.pdf]

HUMAN RIGHTS WATCH
350 Fifth Avenue, 34th Floor
New York, NY 10118-3299
Tel: 212-290-4700
Fax: 212-736-1300
Email: hrwnyc@hrw.org

HUMAN RIGHTS WATCH

www.hrw.org

Kenneth Roth, *Executive Director*
Michele Alexander, *Development & Outreach Director*
Carroll Bogert, *Associate Director*
Emma Daly, *Communications Director*
Barbara Guglielmo, *Finance & Administration Director*
Peggy Hicks, *Global Advocacy Director*
Iain Levine, *Program Director*
Andrew Mawson, *Deputy Program Director*
Suzanne Nossel, *Chief Operating Officer*
Dinah PoKempner, *General Counsel*
James Ross, *Legal & Policy Director*
Joe Saunders, *Deputy Program Director*

Program Directors
Brad Adams, *Asia*
Holly Cartner, *Europe & Central Asia*
David Fathi, *United States*
Georgette Gagnon, *Africa*
José Miguel Vivanco, *Americas*
Sarah Leah Whitson, *Middle East & North Africa*
Joseph Amon, *HIV/AIDS*
John Biaggi, *International Film Festival*
Peter Bouckaert, *Emergencies*
Richard Dicker, *International Justice*
Bill Frelick, *Refugee Policy*
Arvind Ganesan, *Business & Human Rights*
Steve Goose, *Arms*
Liesl Gerntholtz, *Women's Rights*
Scott Long, *Lesbian, Gay, Bisexual & Transgender Rights*
Joanne Mariner, *Terrorism & Counterterrorism*
Lois Whitman, *Children's Rights*

Advocacy Directors
Steve Crawshaw, *United Nations*
Juliette de Rivero, *Geneva*
Jean-Marie Fardeau, *Paris*
Marianne Heuwagen, *Berlin*
Lotte Leicht, *European Union*
Tom Malinowski, *Washington DC*
Tom Porteous, *London*

Board of Directors
Jane Olson, *Chair*
Bruce J. Klatsky, *Vice-Chair*
Sid Sheinberg, *Vice-Chair*
John J. Studzinski, *Vice-Chair*
Louise Arbour
Lloyd Axworthy
David M. Brown
Jorge Castañeda
Tony Elliott
Hassan Elmasry
Michael G. Fisch
Michael E. Gellert
Richard J. Goldstone
Vartan Gregorian
James F. Hoge, Jr.
Wendy Keys
Robert Kissane
Joanne Leedom-Ackerman
Josh Mailman
Susan Manilow
Kati Marton
Linda Mason
Barry Meyer
Pat Mitchell
Joel Motley
Samuel K. Murumba
Catherine Powell
Sigrid Rausing
Victoria Riskin
Shelley Rubin
Kevin P. Ryan
Jean-Louis Servan-Schreiber
Darian W. Swig
John R. Taylor
Shibley Telhami
Catherine Zennström

To:

Dr Jocalyn Clark MSc PhD
Senior Editor
PLoS Medicine
FAX: 416-978-6826

From:

Iain Levine
Program Director
Human Rights Watch
New York, NY

Dr. Clark,

I am writing in response to your request for documentation of Human Rights Watch's approval of research conducted by J. Elizabeth Cohen and Joseph J. Amon related to recently detained injection drug users (IDU) in Guangxi Province, China (manuscript 08-PLME-RA-0726R2).

All researchers who are setting out on field investigations supported by Human Rights Watch are required to submit a pre-mission memo outlining the aims of the research mission; the methodological approach to be taken and likely sources of information. The memo must explain security measures to be taken to protect Human Rights Watch's own staff and consultants, direct collaborators such as translators and fixers, partners on the ground – especially local government and non-governmental officials - and the individuals, especially witnesses to and victims of human rights violations, who provide testimony to our staff – from their association or cooperation with Human Rights Watch. The memo details methods to ensure the anonymity of individuals and protection of the information collected, including the coding of information given and the encrypted storage of sensitive testimony.

The pre-mission memo for the Guangxi IDU project was reviewed by staff in our Asia Division, including experts on China, by our legal and policy department, and by myself, and approved in May 2007, prior to the commencement of field research in July 2007.

In all of our work we put the protection of individuals as our utmost concern and we carefully consider how our public and private statements might impact upon the wellbeing of those we are trying to protect. I appreciate your dedication to ensuring that the research you publish meets the highest standards of protections for individual subjects and can assure you that our internal review and assessment of the ethics of our work and the protection of individual subjects is both thorough and rigorous.

Sincerely,

Iain Levine

Iain Levine
